# Conserved regulators of Rag GTPases orchestrate amino acid-dependent TORC1 signaling

**DOI:** 10.1038/celldisc.2015.49

**Published:** 2016-03-08

**Authors:** Katie Powis, Claudio De Virgilio

**Affiliations:** 1 Department of Biology, University of Fribourg, Fribourg, Switzerland

**Keywords:** Rag GTPases, amino acid signaling, target of rapamycin complex 1

## Abstract

The highly conserved target of rapamycin complex 1 (TORC1) is the central component of a signaling network that couples a vast range of internal and external stimuli to cell growth, proliferation and metabolism. TORC1 deregulation is associated with a number of human pathologies, including many cancers and metabolic disorders, underscoring its importance in cellular and organismal growth control. The activity of TORC1 is modulated by multiple inputs; however, the presence of amino acids is a stimulus that is essential for its activation. Amino acid sufficiency is communicated to TORC1 via the highly conserved family of Rag GTPases, which assemble as heterodimeric complexes on lysosomal/vacuolar membranes and are regulated by their guanine nucleotide loading status. Studies in yeast, fly and mammalian model systems have revealed a multitude of conserved Rag GTPase modulators, which have greatly expanded our understanding of amino acid sensing by TORC1. Here we review the major known modulators of the Rag GTPases, focusing on recent mechanistic insights that highlight the evolutionary conservation and divergence of amino acid signaling to TORC1.

## Introduction

The growth, survival and propagation of all cells and organisms depend on appropriately reacting to fluctuations in their environment. For eukaryotes, cell growth and proliferation are coordinated through a number of finely tuned signaling pathways that sense and respond to diverse environmental cues, such as growth factors, hormones and nutrients. At the center of one of the most extensively studied nutrient-sensing networks lies the target of rapamycin complex 1 (TORC1): a multiprotein complex that couples external and internal stimuli to processes that govern cell growth [1, 2]. The basic architecture and essential functions of TORC1 are conserved throughout the eukaryotic kingdom. In the budding yeast *S. cerevisiae*, TORC1 consists of a dimer of the highly conserved serine/threonine kinase target of rapamycin (TOR), encoded by either *TOR1* or *TOR2*, in addition to Lst8, Kog1 and Tco89 [2]. Mammalian TORC1 also contains a homodimer of TOR (mTOR), the Kog1 ortholog regulatory-associated protein of mTOR (Raptor), mammalian Lst8 (mLst8) and two non-conserved subunits: proline-rich Akt substrate of 40 kDa (PRAS40) and DEP domain-containing mTOR-interacting protein (DEPTOR) [2–4]. Once stimulated, active TORC1 promotes anabolic processes (for example, protein and lipid synthesis) and inhibits catabolic processes (for example, macroautophagy) through the phosphorylation of its downstream effectors. Underlining its pivotal role in cellular and organismal homeostasis, deregulation of TORC1 signaling is linked to numerous human diseases, including many cancers and metabolic disorders [1]. Thus elucidation of the vast and complex network of factors that act upstream of TORC1 remains a significant research goal.

TORC1 can be modulated by a number of different inputs, among which amino acids represent primordial cues that cannot be compensated for by any other stimulus [5–11]. Our understanding of amino acid signaling to TORC1 has increased considerably since the discovery that the highly conserved Rag GTPases are central to this pathway. The Rag GTPases have therefore been the focus of numerous studies in yeast, fly and mammalian model systems, resulting in the elucidation of many additional Rag GTPase modulators. This review outlines the major known regulators of the Rag GTPases, focusing on recent findings that provide insight into the mechanisms of amino acid-dependent TORC1 signaling and their conservation throughout evolution.

## The Rag GTPases

The Ras-related GTP-binding protein (Rag) family of GTPases consists of Gtr1 and Gtr2 in *S. cerevisiae* and RagA, RagB, RagC, and RagD in higher eukaryotes. At the amino acid level, human RagA demonstrates considerable homology with RagB (90% identity) and its ortholog Gtr1 (48% identity). Similarly, RagC shares 81% sequence identity with RagD and 46% with its ortholog Gtr2 [12, 13]. In contrast, the sequence identity shared between RagA/B and RagC/D, or between Gtr1 and Gtr2, is less than 25% [13, 14]. Rag GTPases function as heterodimers in which one monomer of either RagA or RagB partners with one monomer of RagC or RagD [13]. Likewise, Gtr1 forms a heterodimer with Gtr2 [14]. However, complete activation of the Rag heterodimers occurs only when RagA/B or Gtr1 is loaded with GTP and RagC/D or Gtr2 is loaded with GDP [5, 15–17]. This active form of the Rag heterodimer is promoted by the presence of amino acids and is able to directly bind TORC1 via Kog1 (or Raptor in mammals) to stimulate TORC1 activity [5, 15, 17].

In flies and mammals, Rag–Raptor binding promotes the translocation of TORC1 from the cytoplasm to the surface of the lysosome where it is suitably placed to interact with both Rheb, an endomembrane-associated GTPase that activates TORC1 when loaded with GTP [5, 17], and the microspherule protein MCRS1 that stabilizes its interaction with Rheb^GTP^ [18]. In addition to promoting mTORC1 activity, the Rag heterodimer is also important for attenuating it [19, 20]. The inactive form of the heterodimer interacts with the tuberous sclerosis complex (TSC), which consists of TSC1, TSC2 and TBC1D7 [21], and recruits it to the lysosomal membrane where TSC2 acts as GTPase activating protein (GAP) on Rheb [19, 22, 23]. The switch from the active GTP-bound to the inactive GDP-bound form of Rheb allows mTORC1 to be fully released from the lysosome, thereby entirely shutting off mTORC1 signaling upon amino acid withdrawal [19]. Although these findings place TSC2 in an amino acid-sensory branch upstream of TORC1, two parallel studies concluded that it is rather the presence of growth factors/insulin, but not amino acids, that controls the subcellular localization of TSC2 [18, 24]. Additional research is therefore required to clarify whether these discrepancies may originate from the use of different cell types and/or distinct disparities in the experimental setup in these studies.

Although yeast cells do contain a Rheb ortholog, termed Rhb1, there is no evidence to suggest that it is involved in TORC1 signaling. Moreover, TORC1 was found to constitutively localize on the vacuolar membrane, as well as in punctate structures adjacent to the vacuole, in the presence and absence of leucine [15]. Similarly, although the presence of normal levels of TORC1 at the vacuolar membrane required the expression of Gtr1 and/or Gtr2 *per se*, TORC1 also remained detectable at the vacuolar membrane (and within perivacuolar puncta) when wild-type cells were starved for nitrogen [25]. Thus it appears that the mechanism by which the active Gtr1–Gtr2 heterodimer stimulates TORC1 activity following its interaction with Kog1 may differ from that of higher eukaryotes.

## Structure of the Rag GTPases

Considerable insight into the mechanisms of nucleotide-dependent Rag GTPase activation could be derived from the structural resolution of two forms of the Gtr1–Gtr2 heterodimer: partially active Gtr1^GTP^-Gtr2^GTP^ (both bound with the nonhydrolyzable GTP analog GMPPNP) and fully active Gtr1^GTP^-Gtr2^GDP^ [12, 26]. Gtr1 and Gtr2 adopt a similar overall fold, comprising an N-terminal GTPase domain (G domain) and a C-terminal domain (C domain). The Gtr1-Gtr2 heterodimer harbors a pseudo-twofold symmetry, with the majority of its interactions occurring between the two structurally similar C domains within the heterodimeric complex [12, 26]. The C domains of both Gtr1 and Gtr2 contain a roadblock domain, which come together to form a rigid profilin-like fold that may stabilize the complex [12].

Comparisons between the two structures revealed that GTP hydrolysis triggers a large G domain rearrangement [26]. In their GTP-bound forms, the G domains of Gtr1 and Gtr2 are rotated away from each other. However, upon GTP hydrolysis, the G domain of Gtr2 undergoes a 28° rotation that brings it into contact with the G domain of Gtr1 [26]. Although this newly created interaction surface does not directly affect the guanine nucleotide-binding regions of either Gtr1 or Gtr2, the rotation could introduce steric constraints that indirectly affect the binding, dissociation or hydrolysis of the nucleotide associated with the other protein. The G domain of Gtr1 is also predicted to undergo a similar conformational change, as the residues involved in the Gtr2 G-domain rearrangement are highly conserved in Gtr1 [26], raising the possibility that the nucleotide-binding state of one G domain may be able to influence that of the other. The C domain of Gtr2 remains rather rigid; however, it does make numerous interactions with the rotated G domain of Gtr2^GDP^ that are predicted to stabilize the GDP-bound conformation [26].

The dynamic G-domain rearrangements are likely to modulate the interactions between the Rags and their effectors. In both higher eukaryotes and yeast, the active form of the Rag heterodimer interacts more strongly with the TORC1 subunit Raptor (or its ortholog Kog1 in yeast) than the partially active or inactive forms [5, 12, 15, 27]. Importantly, the residues within RagA/B that are involved in the Rag–Raptor interaction are located within the corresponding regions of the Gtr1 G domain that are predicted to undergo substantial rearrangements upon GTP hydrolysis [12, 26]. It is therefore likely that the G domain rotations, resulting from changes in the nucleotide-loading state of the Rag GTPases, expose a binding surface for TORC1 on the heterodimer.

If amino acid-dependent TORC1 signaling is regulated by GTP/GDP charging of the Rag heterodimers, then what mechanisms exist to communicate amino acid availability to the Rag GTPases, and which proteins are involved in the modulation of their nucleotide-binding state? Typically, regulators of GTPases include guanine nucleotide exchange factors (GEFs), which promote the exchange of GDP for GTP; GAPs, which stimulate the hydrolysis of GTP to GDP; and guanine nucleotide dissociation inhibitors (GDIs), which stabilize guanine nucleotide binding. The activity of GTPases may also be influenced by additional factors, such as posttranslational modifications, changes in subcellular localization and/or associations with other proteins, which may be coupled to or independent of those that act on the GTP/GDP-loading state. Indeed, an arsenal of additional proteins, including GEFs, GAPs and GDIs, has recently been identified as regulators of the Rag GTPases.

## The Ego1–Ego2–Ego3 ternary complex (EGO-TC)/Ragulator is required for Rag GTPase function

The first indication that the Rag GTPases require additional proteins to exert their function arose from a genetic screen for mutants that are unable to recover from exposure to rapamycin (named *ego* mutants: *E*xit from rapamycin-induced *G*r*O*wth arrest). Here Gtr2 was shown to form a complex with Ego1 and Ego3 that positively regulates microautophagy during rapamycin recovery [28]. In another study, a complex consisting of Gtr1, Gtr2, Ego1, Ego3 and Ltv1 (termed GSE complex) was initially proposed to be required for sorting the general amino acid permease Gap1 to the plasma membrane in response to amino acid availability [16]. Several additional studies clarified that Gtr1 and Gtr2 form a vacuolar membrane-localized complex with Ego1, Ego3 and the recently identified Ego2 (together termed the EGO complex (EGOC)), and all components are required for amino acid-dependent activation of TORC1 [15, 29, 30].

The EGOC is anchored to the membrane of the vacuole through Ego1, which is N-terminally myristoylated and palmitoylated [15, 31–33]. Although the crystal structure of Ego3 has been solved as both a homodimer and homotetramer [29, 34], the structure of the EGO-TC indicates that only one monomer of each Ego3 and Ego2 associate with vacuolar-anchored Ego1 [30]. Ego2 associates with the C-terminal α-helix of Ego1, while Ego3 makes numerous interactions with the extreme C-terminus of Ego1, which are stabilized by the presence of Ego2. The fully assembled EGO-TC provides a stable platform for the Gtr1–Gtr2 heterodimer on the vacuolar membrane [30]. Both Ego2 and Ego3 contain roadblock domain-like folds that typically form interaction surfaces for GTPases [35] and that are likely to facilitate the recruitment of the Rag heterodimer to the vacuole.

Mammalian Rag GTPases also function together with a larger complex, named Ragulator, which comprises five subunits: LAMTOR1/p18, LAMTOR2/p14, LAMTOR3/MP1, LAMTOR4/C7orf59 and LAMTOR5/HBXIP (LAMTOR: *L*ysosomal *A*daptor and *M*itogen-activated protein kinase and m*TOR* activator/regulator) [36, 37]. Despite weak sequence similarities between any of the component proteins, structural comparisons indicate that Ragulator is likely the mammalian equivalent of the EGO-TC [30]. LAMTOR2 and LAMTOR3 form a heterodimer with each monomer containing a roadblock domain that is structurally similar to that of Ego3 [29]. LAMTOR4 and LAMTOR5 have high structural homology with Ego2 as well as its paralog Ego4, whose role is currently unknown [30, 35]. Finally, similar to Ego1, LAMTOR1 anchors Ragulator to lysosomal membranes via N-terminal lipid modifications [38]. Future analyses of the crystal structure of Ragulator will certainly be helpful in delineating the similarities and differences between the proposed pentameric Ragulator and the heterotrimeric EGO-TC.

Similar to the EGO-TC, Ragulator is also required for the localization of the Rag heterodimer to lysosomal membranes and amino acid-dependent mTORC1 activation [37]. However, Ragulator appears to be more than simply a platform for the Rag GTPases. First, the Ragulator–Rag interaction was enhanced by the absence of amino acids and Ragulator showed a preference for binding inactive nucleotide-free/GDP-bound RagA/B *in vitro* [36]. Second, after purification from cell lysates, the pentameric complex was shown to possess GEF activity toward RagA/B, thereby promoting the exchange of GDP for GTP and consequently the active form of the heterodimer [36]. This mechanism involves the interaction of Ragulator with the conserved vacuolar H^+^-ATPase (v-ATPase), which also resides on the lysosomal membrane [39]. Although amino acid deprivation strengthened the v-ATPase–Ragulator–Rag interaction, neither amino acid levels nor v-ATPase inhibition affected the subcellular localization of the Ragulator or the Rag GTPases. Instead, v-ATPase function was required for Rag GTPases to activate mTORC1 in response to amino acids. *In vitro* cell-free assays with purified lysosomes, Rag proteins and mTORC1 components suggested that the amino acid signal sensed by the v-ATPase is generated from within the lysosome and it is this sensing that modulates the activity of Rag GTPases via its interaction with Ragulator [39]. Interestingly, amino acids were recently shown to modulate the assembly and activity of the v-ATPase in an mTORC1- and phosphoinositide-3 kinase-independent manner. The authors observed increased v-ATPase assembly in amino acid-starved cells (conditions in which the v-ATPase more strongly binds Ragulator), and a reversal of this effect upon amino acid re-addition [40]. Future studies on the mechanism by which amino acids regulate v-ATPase assembly are therefore likely to provide valuable insight into the mechanisms of Ragulator–Rag activation. Of note, the v-ATPase–Ragulator complex has also been found to recruit the scaffold protein AXIN, which causes inhibition of Ragulator GEF activity (toward RagB) and activation of AMPK via AXIN-bound LKB1 at the lysosome specifically when cells were starved for glucose [41].

In addition to genome-wide screens for protein–protein interactions, which have identified associations between several subunits of the peripheral membrane domain of the v-ATPase and both Ego1 and Gtr1 [42, 43], recent evidence suggest that the v-ATPase in yeast also acts upstream of Rag GTPases to control TORC1 [44]. Accordingly, it was proposed that the v-ATPase integrates information regarding the cytosolic pH as a proxy for the quality and quantity of available carbon sources and relays the respective signal to Gtr1/TORC1 [44, 45]. The underlying mechanistic details of how the v-ATPase impinges on Gtr1 remain currently elusive, as it is so far also not known whether the EGO-TC has GEF activity toward Gtr1. Surprisingly, the EGO-TC was dispensable for regulating TORC1 activity during leucine starvation and re-addition when the Gtr1–Gtr2 heterodimer was artificially tethered to the vacuolar membrane, implying that the EGO-TC primarily functions as a scaffold for Gtr1 and Gtr2 on the vacuolar membrane [30].

## Vam6 acts upstream of Gtr1

In one of the first attempts to identify regulators of the Rag GTPases, Binda *et al*. [15] carried out a synthetic dosage lethal screen in yeast cells overproducing a mutant Gtr1 with a reduced affinity for nucleotides (that is, Gtr1^S20L^), thus rendering it inactive. As overexpression of this allele strongly inhibits growth in the absence of wild-type Gtr1, it was reasoned that loss of the Gtr1 GEF would also lead to a similar growth defect. Curiously, the strongest hit, aside from *gtr1∆* itself, was *vam6∆*. Vam6, also known as Vps39, is a member of the class C Vps/HOPS (for *ho*motypic vacuolar fusion and *p*rotein *s*orting) complex that promotes vacuolar fusion events as an effector of the Rab7 GTPase Ypt7 [46–48]. Remarkably, Vam6 (purified from yeast cell extracts) was able to interact with and promote the nucleotide exchange on Gtr1 *in vitro* [15]. Furthermore, similar to the expression of Gtr1^S20L^ allele that is compromised for binding Ego1, loss of Vam6 (but not of Ypt7) abolished the Gtr1–Ego1 interaction and rendered TORC1 unresponsive to cycloheximide treatment, which boosts the levels of free intracellular amino acids as a result of translation elongation inhibition. Combined with epistasis and biochemical analyses in *S. pombe* implying that Vam6 functions upstream of Gtr1/Gtr2 to promote amino acid-dependent TORC1 signaling [49], these findings are highly suggestive of a specific role for Vam6 (alone or combined with additional factors) as a Gtr1 activator following amino acid stimulation.

The vacuole is the major reservoir of amino acids within yeast cells and class C Vps proteins are essential for maintaining proper vacuolar function; thus it is not surprising that cells lacking any of the class C Vps/HOPS proteins exhibit a synthetic sick or lethal phenotype in combination with *tor1* deletion cells, fail to recover from rapamycin-induced growth arrest and exhibit diminished TORC1 activity [50, 51]. However, the Ypt7-independent effects of Vam6 on TORC1 signaling [15] indicate that Vam6 may have additional functions on top of its well-characterized role in vacuolar fusion. It was recently demonstrated that Vam6 has a HOPS complex-independent role in the generation of dynamic contact sites between vacuoles and mitochondria, which facilitate lipid transfer between the two organelles [52, 53]. The assembly and disassembly of these sites, termed vacuolar-mitochondrial patches (vCLAMPs), is controlled by the phosphorylation status of Vam6, which is in turn regulated by metabolic demand [53]. The involvement of Vam6 in controlling HOPS complex-independent processes, in addition to vacuolar fusion, suggest that Vam6 has a central role in balancing a number of vacuolar-related functions with the metabolic requirements of the cell. How the multiple roles of Vam6 impact on each other to achieve cellular homeostasis is certainly worthy of further study.

Although Vam6 is conserved in higher eukaryotes, no association between the mammalian ortholog of Vam6, known as hVps39/hVam6/TLP, and endogenous RagA was detected in HEK-293T cells [36]. Moreover, hVps39 was unable to stimulate GDP or GTP dissociation from RagB *in vitro*, suggesting that this particular mechanism of amino acid sensing may have diverged during evolution [36]. However, similar to the yeast vacuole, proper lysosomal function is essential for TORC1 activation by amino acids, and indeed, knockdown of hVps39 reduced mTORC1 activity [54]. It is also worth noting that mammalian cells contain a second, non-redundant isoform of hVps39, termed hVps39-2/TGFBRAP1/TRAP1, which is a likely component of the human class C core vacuole/endosome tethering (CORVET) complex [55] and whose role in TORC1 signaling has not yet been addressed.

## LeuRS is a conserved positive regulator of Rag GTPases

The branched-chain amino acid leucine is an important activator of TORC1 signaling [56]. Two groups independently demonstrated that balanced levels of cytosolic branched-chain amino acids [57], or of leucine itself [58], are communicated to the Rag GTPases via the tRNA charging enzyme leucyl-tRNA synthetase (LeuRS). The yeast LeuRS Cdc60 was identified as a leucine-dependent Gtr1-interacting partner that positively regulates TORC1 signaling [57]. A combination of coimmunoprecipitation assays, using Gtr1-TAP and an endogenously tagged version of Cdc60, and two-hybrid analyses revealed that LeuRS specifically interacts with Gtr1 (and not Gtr2) and this interaction occurs via the LeuRS editing domain, which is responsible for the recognition and removal of amino acids from mischarged tRNA^Leu^ and undergoes a conformational change when engaged in this activity [59]. A lower level of cytosolic leucine increases the likelihood of tRNA^Leu^ mischarging with alternative branched-chain amino acids (that is, isoleucine and valine), and thus the probability that LeuRS would be engaged in its editing function. It was therefore intriguing that trapping the editing domain in this conformation disrupted its interaction with Gtr1, reduced the amount of GTP-loaded Gtr1 in cells and downregulated Rag-dependent TORC1 signaling in a manner comparable to leucine starvation. These data are consistent with a model in which the Gtr1–LeuRS interaction, which occurs when leucine is abundant, favors the GTP-bound state of Gtr1, perhaps by preventing a negative regulator from acting on Gtr1 or by assisting an activator. Upon leucine deprivation, LeuRS engages in correcting erroneously charged tRNA^Leu^ and dissociates from Gtr1, tipping the balance toward GTP hydrolysis in Gtr1 and inactivation of the heterodimer.

LeuRS was proposed to communicate, via an entirely different mechanism, more directly the availability of leucine to the Rag GTPases in mammalian cells [58]. The authors demonstrated that leucine stimulation recruits LeuRS at lysosomal membranes where it forms a complex with Rags and TORC1. Coimmunoprecipitation experiments using extracts from cells transfected with HA-tagged RagA, RagB, RagC or RagD and purified GST-tagged LeuRS, demonstrated that LeuRS binds exclusively to RagD, preferentially in its GTP-bound form. The authors went on to show that LeuRS enhanced GTP hydrolysis by RagD *in vitro*, thereby proposedly acting as a GAP for RagD and an activator of the Rag heterodimer.

Although both studies convincingly demonstrate that LeuRS is a conserved leucine sensor for the Rag heterodimer, it is difficult to reconcile the underlying mechanistic details. It is possible that the mechanism by which LeuRS regulates Rags has diverged through evolution; indeed, the catalytic arginine in the leucine-binding pocket of mammalian LeuRS, thought to be necessary for its GAP activity, is not conserved in yeast or flies. However, unlike most small GTPases, Gtr2 and RagC/D already contain an arginine in the P-loop of their nucleotide-binding domain predicted to stabilize the transition state, thus a GAP for Gtr2 or RagC/D may not necessarily need to provide a respective arginine [26]. Furthermore, the GAP activity of LeuRS on RagD was not recapitulated in a subsequent study that utilized a method allowing the nucleotide-binding state of each Rag protein in a heterodimer to be selectively monitored [27]. Using this approach, it was demonstrated that the FNIP1/2–Folliculin complex functions as the GAP for RagC and RagD (discussed below) [27]. The possibility that mammalian LeuRS regulates the GDP/GTP-loading status of the Rag heterodimer, analogously to yeast LeuRS, more indirectly, has not been tested yet but may reconcile the discrepancies in the current literature. Identification of the potential negative or positive regulator whose activity is blocked or enhanced, respectively, by the LeuRS–Gtr1 interaction in yeast will be vital to fully comprehend the mechanism by which LeuRS promotes TORC1 signaling and its conservation throughout evolution.

## The FNIP–FLCN/Lst4–Lst7 complexes are conserved Rag GTPase GAPs

Although initial experiments indicated that the nucleotide-binding state of Gtr1 or RagA/B is the main determinant of Rag GTPase activity, Gtr2 or RagC/D must be in its active GDP-bound form to fully activate TORC1. Later, it was shown that the nucleotide-binding state of RagC/D actually dictates the interaction between the Rag heterodimer and the TORC1 subunit Raptor: RagC/D^GDP^ strongly promotes the Rag–Raptor interaction, whereas the presence of RagC/D^GTP^ abolishes it [27].

In 2013, the Folliculin (FLCN) tumor suppressor was identified as a positive regulator of RagC/D in mammals and flies. Two groups reported that FLCN, in a complex with one of its binding partners FNIP1 or FNIP2, is required for the amino acid-induced recruitment of TORC1 to the lysosome and its subsequent activation, suggesting a role for the FNIP1/2–FLCN complex in transducing amino acid signals to TORC1. Both studies went on to show that amino acid starvation promotes the FNIP1/2-dependent recruitment of FLCN to the lysosomal membrane, as well as its interaction with the Rag heterodimer [27, 60]. Consistently, the FNIP1/2–FLCN complex interacted preferentially with the heterodimer when either RagA or RagB contained an inactivating mutation that hindered nucleotide binding [27, 60]. Similar observations were made for FLCN in a third independent study [61]. Although the FNIP1/2–FLCN complex interacted directly with the G domain of RagA (and presumably RagB) [60], the FNIP1/2–FLCN complex did not stimulate GDP dissociation from RagB [27]. Instead, the FNIP1/2–FLCN complex stimulated the GTPase activity of RagC and RagD, which promoted the binding of the Rag heterodimer to Raptor [27]. Based on these data, the simplest model predicts that the FNIP1/2–FLCN complex is driven to the lysosomal membrane in the absence of amino acids, where it interacts with the inactive form of RagA/B. The complex would therefore be in place to promote the active GDP-bound form of RagC/D, via its GAP activity toward RagC/D, when amino acids become available.

The yeast orthologs of FNIP1/2 and FLCN, termed Lst4 and Lst7, respectively, have an analogous role in activating the Rag GTPases [62]. Similar to the findings in mammalian cells, Lst4 and Lst7 form a stable complex in yeast cells and are both essential for fully activating TORC1 signaling upon amino acid stimulation. Furthermore, both proteins shuttle to and from the membrane of the vacuole in amino acid-starved and restimulated cells, respectively. However, whereas the FNIP1/2–FLCN complex associated with the Rag GTPases more strongly in the absence of amino acids, the association between the Lst4–Lst7 complex and Gtr1–Gtr2 was slightly reduced after amino acid starvation and considerably improved after restimulation. Furthermore, the preference for binding the Rag heterodimer when a nucleotide-free, inactive version of RagA/B was expressed was not recapitulated in yeast. However, the Lst4–Lst7 complex preferentially bound GTP-loaded Gtr2 and stimulated its GTP-hydrolytic activity. Thus, in yeast, amino acid starvation drives the recruitment of the Lst4–Lst7 complex to the vacuole where it is poised to interact with the Gtr1–Gtr2 heterodimer. After amino acid restimulation, the Lst4–Lst7 complex associates with inactive Gtr2^GTP^ and exerts its GAP activity, therefore promoting the active state of the heterodimer and TORC1 signaling.

Both models described above require the existence of a signal that communicates the presence of amino acids to the lysosomal- or vacuolar-associated FNIP1/2–FLCN and Lst4–Lst7 complexes, ensuring that RagC/D or Gtr2 is activated at the appropriate time, that is, when cells are restimulated with amino acids. However, the signal(s) would act at different steps in the activation process. In mammalian cells, such a signal would act subsequent to the association of FNIP1/2–FLCN with Rag GTPases at lysosomes and locally stimulate the GAP activity and consecutive release from the lysosomal membrane of the FNIP1/2–FLCN complex. In yeast, the signal would prompt the binding of the Lst4–Lst7 complex to Gtr2, which would then be rapidly followed by the hydrolysis of Gtr2-bound GTP and the release of the Lst4–Lst7 complex from the vacuolar membrane. Thus, a major outstanding question is the identity of the respective signal(s), which could well be different in higher eukaryotes and yeast. If the FNIP1/2–FLCN complex is recruited to lysosomes via nucleotide-free RagA/B in starved cells, it is possible that the amino acid-induced activation of RagA (for instance via Ragulator) and the switch to its GTP-bound form promotes the GAP activity of the FNIP1/2–FLCN complex and subsequent release from the lysosome. However, this cannot be the case in yeast, as neither Gtr1 nor Gtr2 is required for the recruitment of Lst4–Lst7 complex to the vacuole. How the Lst4–Lst7 complex associates with the membrane of the vacuole is not known, although its redistribution from the cytoplasm to the vacuolar periphery after the withdrawal of amino acids is antagonized by the activity of TORC1 itself [62]. Such a negative feedback loop may also exist in higher eukaryotes, as a 1-h treatment of cells with the ATP-competitive TORC1 inhibitor Torin triggered the recruitment of endogenous FLCN to lysosomes [61]. Interestingly, however, Torin was unable to prevent the release of FLCN from lysosomes when cells were restimulated with amino acids [27].

The molecular function of FLCN is of great interest given that loss of function mutations are known to cause the Birt–Hogg–Dubé syndrome, an inherited condition that increases susceptibility to renal carcinomas, pulmonary cysts and fibrofolliculomas [63–66]. The characterization of FLCN as a positive amino acid-induced stimulator of TORC1 activity opens new avenues for research into how the loss of FLCN function results in the tumor formation typical of those affected by Birt–Hogg–Dubé syndrome.

## The SEA/GATOR complexes modulate the activity of the Rag GTPases

Whereas numerous crucial Rag GTPase activators have been identified, the proteins involved in inactivating TORC1 when amino acids are scarce have been less forthcoming. A genome-wide screen in yeast identified the Seh1-associated complex (SEAC) proteins Npr2 and Npr3 as negative regulators of TORC1 [67], but it was only recently that the conserved role of the SEAC in amino acid-dependent TORC1 regulation was fully described [68–70].

SEAC is made up of eight proteins that can be divided into two subcomplexes with opposing activities on TORC1 [69, 70]. Iml1/Sea1, Npr2, and Npr3 are negative regulators of TORC1 signaling and accordingly termed SEACIT (for SEAC inhibiting TORC1). The remaining five members, Seh1, Sec13, Sea2, Sea3, and Sea4, positively regulate TORC1 and are together termed SEACAT (for SEAC activating TORC1). Both subcomplexes exert their effects on TORC1 activity through the Rag GTPases. Following leucine withdrawal, Iml1 transiently interacts with Gtr1 in an Npr2- and Npr3-dependent manner on the vacuolar membrane. By assaying the GTPase activity of Gtr1 *in vitro*, it was demonstrated that Iml1 exerts GAP activity toward Gtr1, stimulating the hydrolysis of its bound GTP to GDP, thus providing the molecular mechanism for the Rag GTPase-dependent TORC1 downregulation. Of note, the assembly of SEACIT depends on Npr2 phosphorylation by a yet unknown protein kinase, which is antagonized by PP2A when the cytosolic methionine levels are sufficiently high to ensure C-terminal methylation and consequently activation of its catalytic subunit [71]. How SEACAT activates Rag-dependent TORC1 signaling is less clear, though genetic experiments have placed SEACAT upstream of SEACIT, suggesting that SEACAT positively regulates TORC1 activity by interfering with the inhibitory activity of Iml1 on the Rag GTPases.

All SEAC members are conserved in higher eukaryotes and, similar to in yeast, can be divided into two subcomplexes. GATOR1 (for GAP activity toward Rags) comprises DEPDC5, NPRL2 and NPRL3 and acts as a GAP toward the Gtr1 homologs RagA and RagB to inactivate mTORC1 in the absence of amino acids. GATOR2, which contains Seh1L, Sec13, Wdr24, Wdr59 and Mios, activates mTORC1 and most likely exerts this function by inhibiting the action of GATOR1 on RagA/B [68].

The proteins that make up SEACIT and GATOR1 contain motifs that are consistent with their function: Npr2 and Npr3 of SEACIT and NPRL2 and NPRL3 of GATOR1 contain roadblock-like longin domains, which are a common feature of GTPase interacting proteins [35, 72], and a region conferring the GAP activity of the SEACIT was identified within Iml1 [69]. Although it has not yet been established whether the Iml1 homolog DEPDC5 provides the GAP activity toward RagA/B in GATOR1, expression of DEPDC5 in yeast partially rescued the TORC1 inhibition defect in cells lacking Iml1, suggesting that it may provide the same activity [69].

Components of SEACAT and GATOR2 are interesting in that they all contain β-propeller-forming WD-40 repeats that are typically found in membrane-coating complexes [72]. Curiously, Sec13 is an established and conserved component of coat protein complex II vesicles and is consequently vital for the function of the secretory pathway. In addition, both Sec13 and Seh1/Seh1L are part of the nuclear pore complex in yeast and mammals. An appealing hypothesis is that nuclear pore complex and/or secretory pathway activity is intimately linked to the activity of TORC1 through the different activities of Sec13 and Seh1 [70]. Exactly how the different cellular functions of SEAC/GATOR proteins affect their role in Rag GTPase regulation will therefore be an attractive question for future studies.

## Ubiquitination of RagA/B recruits GATOR1

A driving force behind the recruitment of GATOR1 to the Rag heterodimer is the K63-linked ubiquitination of RagA [73, 74]. Deng *et al*. [73] demonstrated that RagA is reversibly ubiquitinated on lysines 142, 220, 230 and 244 by the lysosomal membrane-associated RING family E3 ligase RNF152 after the removal of amino acids. Ubiquitinated RagA bound more tightly to GATOR1, providing a convincing mechanism for the inactivation of RagA and downregulation of mTORC1 signaling in amino acid-limiting conditions [73]. However, a second group reported that the level of ubiquitinated RagA is instead elevated when cells were restimulated with amino acids after a period of starvation [74]. Although this discrepancy seems puzzling, it is not inconceivable that the activity of the Rag heterodimer is modulated by several E3 ubiquitin ligases that act upon RagA in different cellular contexts. Indeed, Jin *et al*. [74] also found that RagA was ubiquitinated by Skp2, the F-box component of the Skp1/Cullin/F-box E3 ligase complex, on lysine 15. Skp2-mediated ubiquitination of RagA and recruitment of GATOR1 upon amino acid refeeding was dependent on the activity of mTORC1 and may therefore represent a negative feedback loop to prevent mTORC1 hyperactivation. Supportive of this notion, the authors showed that TORC1 activity diminishes after prolonged amino acid stimulation, indicating that a mechanism must exist to eventually shut off TORC1 activity. Analogous mechanism(s) may also exist in yeast where amino acid stimulation causes transient TORC1 hyperactivation in cells that were pregrown on a poor nitrogen source [75].

Ubiquitination is a conserved mechanism that controls numerous essential regulatory processes in eukaryotic cells. Given the structural and functional conservation between GATOR1 and SEACIT, it is conceivable that ubiquitination also mediates the association of SEACIT with Gtr1. As the interaction between Gtr1 and SEACIT is transiently enhanced after amino acid withdrawal, a mechanism similar to that reported by Deng *et al*. [73], in which a RING family E3 ligase targets Gtr1 in amino acid-limiting conditions, might be more likely to exist. Indeed, the Skp2-targeted K15 is replaced by an arginine in Gtr1, whereas the RNF152-targeted K230 and K244 are conserved. Addressing the role of ubiquitination on the Gtr1–SEACIT interaction will be essential to uncover the degree of conservation between the mechanisms of GATOR1 and SEACIT function.

## Sestrins negatively regulate the Rag GTPases

Several studies have reported that a group of proteins called Sestrins (Sestrins 1–3) also function upstream of the Rag GTPases to negatively regulate TORC1 activity [76–79]. Although the Sestrins do not appear to be conserved in yeast, they are present in worms, flies and mammals where they are induced upon various environmental insults [80]. Sestrins had been previously described to repress mTORC1 signaling, although it was thought that this occurs via AMPK-dependent activation of TSC2 [81]. Now, four groups have independently demonstrated that Sestrins exert their inhibitory function through the Rag GTPase-dependent branch of mTORC1 activation.

Peng *et al*. [76] reported that Sestrins directly bind to the Rag proteins and prevent GDP dissociation from RagA/B, thus inactivating the heterodimer. Supportive of this notion, the authors also identified a conserved putative GDI motif within the amino acid sequence of Sestrins. At the same time, several additional studies demonstrated that Sestrins interact with GATOR2 and function upstream of GATOR1 to inhibit mTORC1 activity [77–79]. Parmigiani *et al*. [77] and Chantranupong *et al*. [78] both reported that Sestrin2 (and, to a lesser extent, Sestrin1 and Sestrin3) interact with GATOR2 in a manner that was enhanced upon amino acid deprivation. In both studies, Sestrin2 inhibited mTORC1 activity in a GATOR1- and Rag-dependent manner, without affecting the GATOR1–GATOR2 and GATOR1–Rag interactions, or the nucleotide-loading state of the Rags [77, 78]. In contrast, Kim *et al*. [79] found that high levels of Sestrin2 (arising from stress-induction or ectopic expression) diminished the interaction between GATOR2 and GATOR1 in a dose-dependent manner, while simultaneously increasing the GATOR1–RagB interaction and the cellular level of inactive, GDP-loaded RagB.

These studies raise numerous interesting questions. Is it possible that different inputs influence the mechanism by which Sestrins and GATOR inactivate the Rag heterodimer? Kim *et al*. [79] have speculated that Sestrin2 drives the displacement of GATOR2 from GATOR1–RagB only when it is vastly overproduced, as it is when cells are exposed to particular environmental stressors, such as genotoxic, oxidative or endoplasmic reticulum stress. Under conditions of amino acid withdrawal, which does not upregulate Sestrins at the transcriptional level, the moderate amount of Sestrin2 may impact upon GATOR and the Rag GTPases via an alternate mechanism [79]. Intriguingly, in this context, Wolfson *et al*. [82] reported that leucine (and less potently isoleucine and methionine) disrupts the Sestrin2–GATOR2 (and similarly the Sestrin1–GATOR2, but not the Sestrin3–GATOR2) interaction by directly binding to Sestrin2. Sestrin2 (and likely Sestrin1) therefore acts as a cytosolic leucine sensor upstream of the GATOR–Rag GTPase–TORC1 branch [82]. The finding that Sestrins may also function as GDIs for RagA/B in amino acid-limiting conditions is a little more difficult to integrate into such a model, specifically also because part of the proposed GDI motif is not exposed at the surface of Sestrin2 in a recently solved crystal structure of Sestrin2 in complex with leucine [83]. However, it can not currently be excluded that Sestrins may regulate amino acid signaling to mTORC1 via a dual mechanism: functioning as GDIs for RagA/B (in amino acid-deprived cells) as well as acting through the GATOR complex to regulate the activity of the Rag heterodimer.

Although the exact mechanism through which Sestrins act on Rag GTPases requires further elucidation, several studies demonstrated that the function of Sestrins in amino acid sensing to TORC1 is physiologically significant. Increasing or diminishing the level of *Drosophila* Sestrin resulted in developmental abnormalities and autophagy defects in flies, which could be suppressed by rendering GATOR components inactive [79]. Mice lacking all three Sestrin-encoding genes do not survive the neonatal period and exhibit constitutively active mTORC1 signaling in the liver, heart and skeletal muscle during neonatal fasting [76]. The sensitivity of mTORC1 signaling to amino acids was significantly reduced in mouse embryonic fibroblasts derived from the triple knockout animals. In these cells, increased mTORC1 activity persisted long after the withdrawal of amino acids but remained dependent on the presence of growth factors [76]. These findings highlight the importance of Sestrins for the physiological response to amino acid starvation and will undoubtedly aid future efforts to unravel the mechanisms underlying the diverse metabolic- and age-related pathologies associated with loss of Sestrin function [84].

## Other modulators

**Figure 1:**
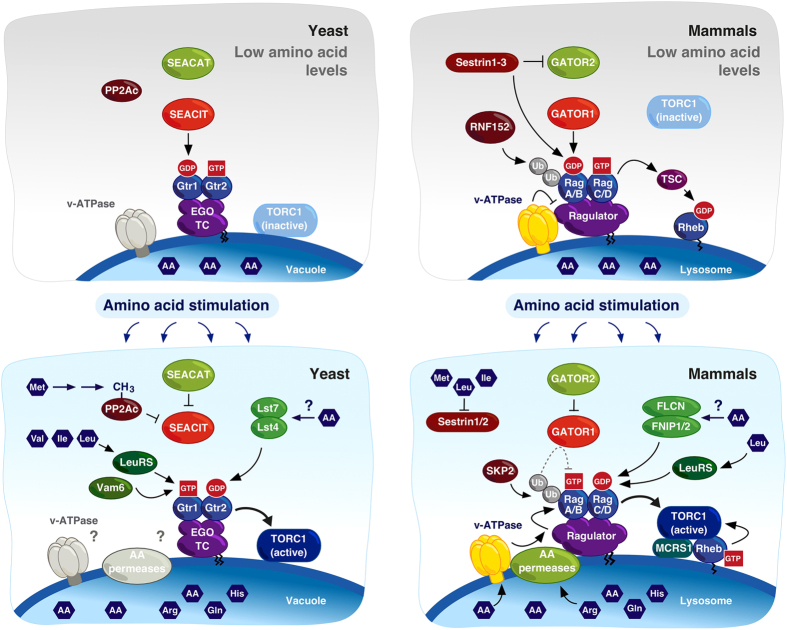
Amino acid-dependent target of rapamycin complex 1 (TORC1) activation by key modulators of the Rag GTPases in yeast and mammals. The activation of TORC1 by amino acids occurs via the Rag GTPases, which are regulated by their guanine nucleotide-loading status. In yeast, TORC1 is constitutively found on the vacuolar membrane and is stimulated when the Rag heterodimer is in its active Gtr1^GTP^-Gtr2^GDP^ conformation (left). In mammals, mTORC1 is localized diffusely throughout the cytoplasm and is recruited to lysosomes by the active RagA/B^GTP^-RagC/D^GDP^ heterodimer, where it can be activated by GTP-loaded Rheb within an MCRS1-stabilized complex (right). In amino acid-starved cells, the Rag heterodimer is maintained in its inactive form by the GTPase activating protein (GAP) activity of either (SEACIT toward Gtr1 in yeast or GATOR1 toward RagA/B in mammals. The GATOR1–RagA/B interaction is driven by RNF152-dependent ubiquitination of RagA/B. Sestrins also contribute to the inactivation of the Rag heterodimer by inhibiting GATOR2 and/or preventing the release of GDP from RagA/B. Furthermore, the inactive Rag heterodimer contributes to the inactivation of Rheb (and thus mTORC1) through the recruitment of the tuberous sclerosis complex (TSC) complex that exerts GAP activity toward Rheb. Upon amino acid stimulation, positive regulators of the Rag GTPases stimulate the formation of the active form of the Rag heterodimer. In yeast, the activity of LeuRS and Vam6 on Gtr1 and Lst4-Lst7 on Gtr2 promote the formation of Gtr1^GTP^-Gtr2^GDP^, while SEACAT interferes with the inhibitory effect of the Gtr1 GAP, SEACIT. In mammalian cells, v-ATPase stimulates the guanine nucleotide exchange factor (GEF) activity of the Ragulator to promote GTP loading of RagA/B, while the FNIP1/2-FLCN complex, and possibly LeuRS, exert GAP activity toward RagC/D, resulting in GDP-loaded RagC/D. GATOR2 also impedes the activity of the RagA/B GAP, GATOR1. Hyperactivation of mTORC1 after prolonged amino acid stimulation may be prevented by Skp2-mediated ubiquitination of RagA/B, which drives the recruitment of GATOR1 to the Rag heterodimer (indicated with a dashed line). The focal points where amino acids directly feed into the Rag GTPase network currently include (i) LeuRS that mediates balanced levels of the branched-chain amino acids leucine, isoleucine and valine to Gtr1 in yeast or leucine levels to RagC/D in mammals, (ii) lysosomal amino permeases that transport and, via still poorly defined mechanisms, signal arginine and/or glutamine (SLC38A9), small unbranched amino acids (SLC36A1/PAT1 (proton-assisted amino acid transporter 1)), and possibly histidine (SLC15A4) levels to the Rag GTPases, (iii) mammalian Sestrin1 and Sestrin2 that dissociate from GATOR2 when directly bound to leucine or to the lower-affinity substrates methionine and isoleucine and (iv) yeast S-adenosylmethionine synthetase and Ppm1 methyltransferase that channel methionine (tandem arrows) into the methylation of the catalytic PP2A subunit (PP2Ac), which antagonizes SEACIT assembly. Whether the v-ATPase or any of the vacuolar membrane-resident amino permeases mediate amino acid signals toward the Rag GTPases in yeast is currently not known (which is why they are depicted in grey in the yeast model on the left). How amino acids impinge on Lst4-Lst7 and FNIP1/2-FLCN is currently not known. Arrows and bars impinging on Rag GTPases denote mechanisms that favor and antagonize, respectively, the indicated nucleotide-binding state. For all other proteins or protein complexes, arrows and bars denote positive and negative interactions, respectively. AA, amino acids; Arg, arginine; Gln; glutamine; His, histidine; Ile, isoleucine; Leu, leucine; Met, methionine; Val, valine. For further details, see text.

In addition to those described above (see Figure 1 for a graphical summary), other modulators of Rag GTPases have been identified so far exclusively in studies of higher eukaryotes (Table 1). Among these, the recent discovery that the lysosomal membrane-localized glutamine/arginine transporter SLC38A9 directly communicates amino acid levels to the Rag GTPases to regulate mTORC1 activity signifies an exciting advance in the search for amino acid sensors [85–87]. SLC38A9 interacts with the Rag GTPases in an amino acid-dependent manner, and disruption of the SLC38A9 gene reduced TORC1 activity in response to arginine but not leucine [85–87]. This finding raises the possibility that there may be other amino acid transporters that are able to directly communicate the availability of specific amino acids to the Rag GTPases. Indeed, another lysosomal membrane-resident, small unbranched amino acid transporter, proton-assisted amino acid transporter 1 (PAT1)/SLC36A1 [88], was previously shown to be important for amino acid-dependent activation of TORC1 in mammals and flies, and interestingly also associates with Rag GTPases [89]. Similarly, the lysosomal membrane-localized histidine transporter SLC15A4 has also been found to mediate mTORC1 activation as part of the cellular response to inflammation, although it remains to be studied whether this process also implicates Rag GTPases [90]. The yeast vacuolar membrane is also home to a number of amino acid transporters, including Avt1-7, Vba1-5, Atg22 and Ers1, which exhibit specificity for a range of different amino acids [91]. Curiously, the transporters most closely related to mammalian SLC38A9 and PAT1 belong to the Avt family of amino acid permeases, whose members are also responsible for controlling the flux of the potent TORC1 activator glutamine across the vacuolar membrane [92, 93]. Evaluating whether one or more of these amino acid transporters can also directly regulate Rag GTPases and TORC1 in a genetically tractable organism such as yeast would greatly accelerate our understanding of this novel aspect of amino acid sensing.

Modulators of Rag GTPases that have been identified so far exclusively in higher eukaryotes finally include the Ste20-related MAP4K3 protein kinase that regulates TORC1 and binds Rag GTPases [94–96], p62 that favors the assembly of the active Rag GTPase-mTORC1 module [97], SH3BP4 (SH3 (Src homology 3 domain)-binding protein 4) that associates with the inactive Rag GTPase complex to inhibit its conversion to the active form [98], c17orf59 that prevents Rag GTPases from binding Ragulator [99], and glutaminase and glutamate dehydrogenase that drive glutaminolysis to promote RagB GTP-loading and consequently mTORC1 activation by unknown mechanisms [100] (Table 1). It will be interesting to understand how these additional modulators fit into the Rag GTPase regulatory network outlined above and whether some of the underlying mechanisms may represent ancestral modes of Rag GTPase control.

It is worth noting that Rag GTPase-independent pathways for amino acid-induced TORC1 activation also exist [101–103]. For instance, sustained TORC1 activity in yeast was reported to depend on a Rag GTPase-independent mechanism that requires the conversion of nitrogen sources to glutamine [75]. In addition, mammalian cells lacking RagA and RagB were still able to respond to glutamine, although not leucine, through a mechanism that required the lysosome, the v-ATPase and the small GTPase Arf1 [101]. The small GTPase Ypt1 and its mammalian paralog Rab1A were also reported to be essential for Rag-independent amino acid-induced activation of TORC1 in yeast and mammalian cells, respectively [102]. Intriguingly, Rab1A was reported to activate TORC1 at the Golgi, suggesting that intracellular organelles other than lysosomes might also have a role in amino acid sensing to TORC1 [102].

## Concluding remarks

The remarkable conservation of the mechanisms that regulate the Rag GTPases underscores their physiological importance across the eukaryotic kingdom. Indeed, mutations in many of the proteins now recognized as conserved components of the amino acid sensing branch of TORC1 signaling are associated with human pathologies. For instance, a mutation that reduces the protein levels of the Ragulator subunit LAMTOR2 causes a primary immunodeficiency syndrome that results in severe growth defects [104]. It was later confirmed that cells isolated from patients carrying this mutation exhibit low mTORC1 activity [37]. Other components of the amino acid sensing branch of mTORC1 activation have been linked to tumor progression. The tumor suppressor FLCN was surprisingly identified as a positive regulator of TORC1 signaling by amino acids [27, 60]. Precisely how loss-of-function mutations in FLCN lead to tumor development requires further investigation, although it is possible that cells attempt to compensate for the reduction in FLCN-mediated mTORC1 activation by amplifying the activity of other mTORC1 activators or growth-promoting pathways [27]. Inactivating mutations in GATOR1 components DEPDC5 and NPRL2 have also been identified in glioblastomas and ovarian cancers [68], and reduced expression of NPRL2 has been detected in a number of other cancers [105–110]. Finally, mutations in DEPDC5 have been recently identified in numerous cases of familial focal epilepsy [111–116]. Our accumulating knowledge of amino acid signaling to TORC1 gleaned from studies in yeast, fly and mammalian model systems will undoubtedly pave the way for future research into prospective therapeutics for these pathologies.

Despite making considerable progress in recent years, many aspects of amino acid signaling to TORC1 have not yet been resolved. LeuRS remains the only conserved sensor of amino acid availability identified so far, although it is expected that there are additional conserved factors that sense the presence of other amino acids. As well as cytosolic sensors, it is likely that the lysosome/vacuole also has a pivotal role in this activity. In mammals, both the v-ATPase and the lysosomal membrane-resident glutamine/arginine transporter SLC38A9 have a role in transducing amino acid signals to Rag GTPases [39, 85, 86]. In yeast, the vacuolar-associated Vam6 activates the Rag GTPases and perturbations in vacuolar function disrupt TORC1 signaling [15, 50]. Another critical outstanding issue concerns how all of the regulatory mechanisms that impinge on the Rag GTPase module are coordinated and integrated. Exactly how do the Rag GTPases respond to multiple inputs (including, besides different amino acids, likely also other nutrient cues such as glucose [41, 117]), and how does this affect TORC1 activation? For instance, some amino acids appear to be more potent TORC1 activators than others, so how do the Rag GTPases and/or their modulators discern them? Is the asymmetrically loaded Rag heterodimer itself self-regulating, that is, can the nucleotide-loading state of one Rag monomer influence that of the other? Understanding the interplay between the various components that affect the activity of the Rag GTPases will vastly extend the current view of amino acid sensing by TORC1 and will be invaluable for the development of novel approaches to combat mTORC1-related diseases.

## Figures and Tables

**Table 1:** Rag GTPase signaling network components in *S. cerevisiae* and mammalian cells

*S. cerevisiae*	*Mammalian cells*	*Description*
Gtr1	RagA, RagB	Rag GTPases, heterodimerize with Gtr2 or RagC/D, respectively, bind to and promote the activity of TORC1 when loaded with GTP [5, 15, 17]
Gtr2	RagC, RagD	Rag GTPases, heterodimerize with Gtr1 or RagA/B, respectively, bind to and promote the activity of TORC1 when loaded with GDP [5, 15, 17]
EGO-TC (Ego1-3)	Ragulator (LAMTOR1-5)	Complexes that provide a platform for the Rag GTPases on the vacuolar/lysosomal membrane [15, 30, 36]; Ragulator also has GEF activity toward RagA/B [36]
v-ATPase	v-ATPase	Stimulates the GEF activity of the Ragulator [39]; binds and mediates pH signals to Gtr1 [44]
Vam6 (Vps39)	hVps39/hVps39-2?	Binds to and promotes the exchange of GDP for GTP in *S. cerevisiae* Gtr1 [15]; acts upstream of and binds Gtr1 in an amino acid-dependent manner *in vivo* in *S. pombe* [49]; hVps39 is neither a RagA/B GEF nor an interacting protein [36]
Cdc60 (Leucyl-tRNA synthetase)	LARS (Leucyl-tRNA synthetase)	Conserved positive regulator of Rag GTPases, communicates balanced levels of branched-chain amino acids in yeast or leucine levels in mammals [57, 58]; binds Gtr1 and promotes its activation or antagonizes its inactivation in yeast [57]; binds to and stimulates [58] or not [27], GTPase activity of RagD
Lst4-Lst7	FNIP1/2-FLCN	Conserved positive regulatory complex of the Rag GTPases and TORC1 signaling, exerts GAP activity toward Gtr2 or RagC/D [27, 60]
SEACIT (Iml1/Sea1, Npr2 Npr3)	GATOR1 (DEPDC5, NPRL2, NPRL3)	Conserved negative regulatory complex of the Rag GTPases and TORC1 signaling, exerts GAP activity toward Gtr1 or RagA/B [68–70]
SEACAT (Seh1, Sec13, Sea2, Sea3, Sea4)	GATOR2 (Seh1L, Sec13, Wdr24, Wdr59, Mios)	Conserved positive regulatory complex of Rag GTPases and TORC1 signaling, likely to function upstream of SEACIT/GATOR1 [68–70]
E3 ligases(s)?	RNF152	RING family E3 ligase, ubiquitinates RagA to promote its interaction with GATOR1, inactivates TORC1 signaling in the absence of amino acids [73]
Skp2?	Skp2	F-box component of the Skp1/Cullin/F-box (SCF) E3 ligase complex, ubiquitinates RagA to promote its interaction with GATOR1, inactivates TORC1 signaling after prolonged exposure to amino acids [74]
?	Sestrin1-3	Negative regulators of Rag GTPases, interact with GATOR2 and inhibit TORC1 activity upstream of GATOR1 and the Rag GTPases [77–79]; may also act as GDIs for RagA/B [76]; Sestrin2 directly binds leucine [82, 83]
Vacuolar amino acid permeases?	SLC38A9	Low affinity arginine/glutamine transporter on the lysosomal membrane, interacts with Rag-Ragulator in an amino acid-dependent manner and stimulates amino acid activation of TORC1 [85–87]
Vacuolar amino acid permeases?	SLC36A1/PAT1	Regulates H^+^-dependent amino acid efflux from the lysosome, associates with the Rag GTPases, required for recruitment of TORC1 to the lysosome in the presence of amino acids [89]
Ste20 family kinase(s)?	MAP4K3	Regulated by amino acids, functions upstream of Rag GTPases to positively regulate TORC1 in response to amino acids [94–96]
?	p62	Adaptor protein that interacts with and stabilizes the active form of Rag GTPase heterodimers, possible alternative platform for the Rag GTPases on the lysosome [97]
?	SH3BP4	Prevents GTP binding to RagB, downregulates TORC1 activity [98]
?	c17orf59	Prevents Rag GTPases from binding Ragulator [99]
? /Gdh1-3	GLS/GDH	Glutaminase (GLS) and glutamate dehydrogenase (GDH) drive glutaminolysis to favor RagB GTP-loading [100]; *S. cerevisiae* expresses no GLS ortholog

Abbreviations: GDI, guanine nucleotide dissociation inhibitor; GEF, guanine nucleotide exchange factor; PAT1, proton-assisted amino acid transporter 1; SEACAT, Seh1-associated complex activating TORC1; SEACIT, Seh1-associated complex inhibiting TORC1; TORC1, target of rapamycin complex 1.
